# Clinical Diversity in Biliary Pancreatitis — Classification of Two Types

**DOI:** 10.1155/1993/13505

**Published:** 1993

**Authors:** Masatoshi Isogai, Kitao Hachisuksa, Akihiro Yamaguchi, Satoshi Nakano

**Affiliations:** 1 Department of Surgery Ogaki Municipal Hospital 4–86, Minaminokawa Ogaki 503 Japan; 2 Department of Gastroenterology Ogaki Municipal Hospital 4–86, Minaminokawa Ogaki 503 Japan

## Abstract

One hundred and seven patients with biliary pancreatitis undergoing operation from 1976 to 1989 were reviewed. To clarify the reason for failure to respond to conventional supportive therapy, 73 patients (68%) who underwent emergency surgery were retrospectively divided into two groups according to the severity of the pancreatitis evaluated at laparotomy and compared. Sixty-two had minimal or mild pancreatitis (Group I), among whom 44 (71%) had life-threatening acute biliary tract disease. All underwent biliary surgery and 4 (6%) subsequently died, 2 due to acute obstructive suppurative cholangitis. Eleven had hemorrhagic necrotizing pancreatitis (Group II), among whom 7 had complications of acute pancreatitis such as pancreatic ascites or abscess. These underwent pancreatic and/or biliary surgery and 3 (27%) died of multi-organ failure.

There appears to be two types of biliary pancreatitis refractory to conventional supportive therapy, which differ in the extent of surgery required and in mortality: (1) minimal or mild pancreatitis with persistent life-threatening acute biliary tract disease (biliary type), and (2) more severe pancreatitis (pancreas type) early in the course of the disease.


*HPB Surgery*, 1993, Vol. 6, pp. 263-276
Reprints available directly from the publisher
Photocopying permitted by license only
© 1993 Harwood Academic Publishers GmbH
Printed in the United States of America

### CLINICAL DIVERSITY IN BILIARY PANCREATITIS — CLASSIFICATION OF TWO TYPES

MASATOSHI ISOGAI, KITAO HACHISUKSA and AKIHIRO YAMAGUCHI

*Department of Surgery, Ogaki Municipal Hospital, 4–86, Minaminokawa, Ogaki, 503, Japan*

SATOSHI NAKANO

*Department of Gastroenterology, Ogaki Municipal Hospital, 4–86, Minaminokawa, Ogaki, 503, Japan*

*(Received 1 August 1992)*

One hundred and seven patients with biliary pancreatitis undergoing operation from 1976 to 1989 were reviewed. To clarify the reason for failure to respond to conventional supportive therapy, 73 patients (68%) who underwent emergency surgery were retrospectively divided into two groups according to the severity of the pancreatitis evaluated at laparotomy and compared. Sixty-two had minimal or mild pancreatitis (Group I), among whom 44 (71%) had life-threatening acute biliary tract disease. All underwent biliary surgery and 4 (6%) subsequently died, 2 due to acute obstructive suppurative cholangitis. Eleven had hemorrhagic necrotizing pancreatitis (Group II), among whom 7 had complications of acute pancreatitis such as pancreatic ascites or abscess. These underwent pancreatic and/or biliary surgery and 3 (27%) died of multi-organ failure.

There appears to be two types of biliary pancreatitis refractory to conventional supportive therapy, which differ in the extent of surgery required and in mortality: (1) minimal or mild pancreatitis with persistent life-threatening acute biliary tract disease (biliary type), and (2) more severe pancreatitis (pancreas type) early in the course of the disease.

KEY WORDS: Biliary pancreatitis, gallstone pancreatitis, acute obstructive suppurative cholangitis, severe pancreatitis

### INTRODUCTION

The timing of surgery for patients with biliary pancreatitis remains controversial. Three different approaches, including early operation[1,2], delayed surgery[3] and expectant[4–7], flexible[8], or selective[9] approaches, are currently being advocated. In our hospital, almost all patients with biliary pancreatitis who had failed to improve with conventional supportive therapy had undergone surgery immediately, provided the patients had presented a reasonable surgical risk. This provided the opportunity to review a substantial number of patients who underwent emergency operation. Blamey *et al.*[10] classified pancreatitis as clinically severe if the patient underwent surgery because of failure of the pancreatitis to resolve. The majority of our emergency surgery patients, however, had minimal or mild pancreatitis at

Address correspondence to: Masatoshi Isogai, M.D., Department of Surgery, Ogaki Municipal Hospital, 4–86, Minaminokawa, Ogaki, 503, Japan laparotomy. It was reported that 13–61%[4,5,7] of patients undergoing surgery within a few days of an attack had no significant evidence of pancreatitis.

The question arises as to what is the critical factor determining the timing of surgery in biliary pancreatitis. The purpose of this study is to clarify the reason for failure to improve on conventional supportive therapy and to discuss the timing and type of surgical intervention for patients with biliary pancreatitis.

### MATERIALS AND METHODS

During the 14 years from January 1976 to December 1989, 2971 consecutive patients with gallstone disease underwent biliary surgery at the Department of Surgery, Ogaki Municipal Hospital. A retrospective review was performed of 107 patients (3.6%) who were diagnosed as having biliary pancreatitis. These included 45 men and 62 women. Their ages ranged from 3 to 87 years (mean, 58 years). The 3-year-old child had a congenital dilatation of the biliary tract. The diagnosis of acute pancreatitis was made on the basis of clinical symptoms of severe abdominal pain and physical signs of upper abdominal tenderness, as well as laboratory evidence of hyperamylasemia. Gallstones were demonstrated by typical ultrasound (US) findings. Laboratory evidence of serum transaminase elevation was used as a diagnostic criteria for assessing gallstones as the cause of pancreatitis[11-13]. Gallstones were confirmed in each case at operation. Other imaging studies such as percutaneous transhepatic cholangiography (PTC), endoscopic retrograde cholangiography (ERC) and computed tomography (CT) were used infrequently to resolve specific questions about ductal or pancreatic anatomic features. Cases involving alcohol ingestion preceding the onset of symptoms or those that had other potential causes of serum transaminase elevation such as acute viral hepatitis, heart failure or drug abuse were excluded from this study. The mean value of serum amylase, glutamic oxaloacetic transaminase (SGOT), glutamic pyruvic transaminase (SGPT) and total bilirubin (T. Bil.) on the day of admission were 1.202 Caraway U. (normal $<135$ C.U.), 354 Karmen U. (normal $<40$ K.U.), 267 Karmen U. (normal $<35$ K.U.) and 3.5 mg/dl (normal $<1.2$ mg/dl), respectively.

The one hundred and seven study patients included: (1) 73 patients (68%) who underwent emergency surgery within 48 hours of admission, and (2) 34 patients (32%) who underwent initial non-operative management followed by delayed surgery during the same hospitalization after an average time of 14 days from admission, when the acute symptoms had subsided.

The majority of the 73 emergency surgery patients showed no objective improvement in response to conventional supportive therapy such as withholding oral feeding, nasogastric suction, volume replacement, antibiotics, and analgesics. Endoscopic sphincterotomy (ES) for impacted ampullary stones was unsuccessful in 2 cases. Deterioration of their general condition, peritonitis and/or sepsis were the important factors determining emergency operation. In some patients, the attending physician opted for emergency operation without initial conservative management.

In the 34 delayed surgery patients, signs and symptoms abated with a rapid decline in the elevated amylase and transaminase levels either after conventional supportive therapy (32 cases), or after ES for impacted ampullary stones (2 cases). In the 32 patients, stones might have passed through, with rapid regression of the disease. In these patients, biliary surgery could be performed safely at any time, without fatalities.

For the purpose of this study, the 73 emergency surgery patients were divided into two groups according to the severity of the pancreatitis evaluated at laparotomy as follows: (1) 62 patients with minimal or mild pancreatitis (Group I), and (2) 11 patients with hemorrhagic necrotizing pancreatitis (Group II). In the 62 Group I patients, the pancreas was reported to be almost normal (26 cases), indurated (4 cases) or edematous (32 cases). In the 11 group II patients, 7 had complications of pancreatitis including intraabdominal abscess (2 cases), pancreatic ascites (4 cases), or intraabdominal abscess and pancreatic ascites (1 case). Three patients had extended pancreatic necrosis.

An analysis of the clinical features, operative findings and outcome of therapy was made and the two groups were compared. Mortality was defined as death occurring any time during the patient's hospitalization.

Data were expressed as mean $\pm$SD. Either the chi-square test with Yates' correction or the Student's $t$ test was used for statistical analysis. Differences were considered significant at $p < 0.05$.

### RESULTS

### *Clinical Features*

There were 30 men and 32 women aged 23 to 87 years old (mean, 59 years) among the 62 Group I patients, while there were 5 men and 6 women aged 37 to 77 years old (mean, 61 years) among the 11 Group II patients. Time between onset of symptoms and operation was 1–13 days (mean, 2.1 days) in Group I, and 1–5 days (mean, 2.0 days) in Group II. No significant differences were noted between the two groups with regard to age, sex and time between onset of symptoms and operation. There were also no significant differences between the two groups with regard to the values of serum amylase, SGOT, SGPT, T. Bil. and white blood cell count (WBC) on the day of admission (Table 1). Charcot's triad (abdominal pain, fever and jaundice) and paralytic ileus due to peritonitis were present in 21 (34%) and 5 (8%) of the 62 Group I patients, and in 0 and 5 (45%) of the 11 Group II patients, respectively (significantly different,, Charcot's triad, $p < 0.05$; paralytic ileus, $p < 0.01$). Four patients (6%) of Group I and 2 (18%) of Group II were in shock on admission, respectively.

### *Operative Findings*

Impacted ampullary stones, floating bile duct stones and gallstones in the gallbladder alone were found in 35 (56%), 13 (21%) and 14 (23%) of the 62 Group I patients, compared with 1 (9%), 2 (18%) and 8 (73%) of the 11 Group II patients, respectively (significantly different, $p < 0.01$).

Acute suppurative cholangitis (ASC) which we defined as presence of purulent bile in the bile duct was found in 26 (42%) of the Group I patients and in 1 (9%) of the Group II patients (significantly different, $p < 0.05$).

Massive accumulation of bile in the abdomen due to acute cholecystitis was found in 5 (8%) of the group I patients and massive pancreatic ascites was present in 5 (45%) of the Group II patients.

**Table 1** Laboratory data on admission

| | *Group I (62 cases)* | *Group II (11 cases)* |
|---|---|---|
| S-Amylase (C.U. N<135) | 1182±957 | 1535±888 |
| SGOT (K.U. N<40) | 362±307 | 243±233 |
| SGPT (K.U. N<35) | 279±214 | 154±104 |
| T.bilirubin (mg/dl N<1.2) | 4.0±2.2 | 2.2±1.7 |
| WBC ($\times 10^3$ cells/$mm^3$) | 12.9±5.0 | 12.7±5.0 |

Values are presented as mean±SD.
SGOT: serum glutamic oxaloacetic transaminase; SGPT: serum glutamic pyruvic transaminase; WBC: white blood cell count.

Distention of the gallbladder and the bile duct was found at operation in almost all Group I patients. However, the intensity of the gallbladder inflammation was slight in most cases. Gangrenous and phlegmonous or edematous cholecystitis were found in 4 (6%) and 3 (5%), respectively, and the remaining 55 pateints (89%) showed an almost normal gallbladder. In Group II patients, gangrenous cholecystitis was observed in 1 patient (9%) and the remaining 10 patients (91%) showed an almost normal gallbladder.

Severe inflammatory biliary diseases encountered at operation in the 62 Group I patients are summarized in Table 2. Severe inflammatory biliary diseases such as ASC,bile peritonitis and gangrenous cholecystitis were present in 20 (57%) of the 35 patients with impacted ampullary stones,in 5 (38%) of the 13 patients with floating bile duct stones and in 4 (29%) of the 14 patients with gallstones in the gallbladder alone. Four patients who had both impacted ampullary stones and ASC were in shock (acute obstructive suppurative cholangitis,AOSC[14]), two of whom had bile peritonitis also.Accordingly, persistent life-threatening acute biliary tract disease including impacted ampullary stones, ASC, AOSC, bile peritonitis and gangrenous cholecystitis were present in 44 (71%) of the 62 Group I patients.

**Table 2** Severe inflammatory biliary diseases in 62 Group I patients

| | *Impacted* stones (35 cases)* | *Floating** stones (13 cases)* | *Gallbladder stones only (14 cases)* |
|---|---|---|---|
| (1) ASC | 16<2> | 4 | 1 |
| (2) ASC + Bile peritonitis | 4<2> | 1 | 0 |
| Serous cholecystitis | 3<1> | 1 | |
| Gangrenous cholecystitis | 1<1> | 0 | |
| (3) Gangrenous cholecystitis | 0 | 0 | 3 |
| Total | 20 (57%) | 5 (38%) | 4 (29%) |

ASC: acute suppurative cholangitis (purulent bile in the common bile duct).
<>: number of patients who were in shock on admission (acute obstructive suppurative cholangitis).
*: at the ampullary region.
**: in the common bile duct.

### *Operative Procedures and Outcome of Therapy*

*Sixty-two patients with minimal or mild pancreatitis (Group I):*
Operation was limited to the biliary tract in 60 patients (96.8%). Of these, 4 underwent cholecystectomy alone.Fifty-six patients underwent bile duct exploration in addition to cholecystectomy. Bile duct exploration was indicated when bile duct stones were found on preoperative PTC or ERC, on routine intraoperative cholangiography or when there was no flow of contrast into the duodenum. Other laboratory abnormalities or operative findings such as cholangitis or bile duct dilatation occasionally prompted bile duct exploration. Sphincterotomy was necessary to remove impacted ampullary stones in 30 cases. The remaining two (3.2%) Group I patients who had edematous pancreatitis received pancreatic drainage in addition to the biliary surgery of cholecystectomy and bile duct exploration.

Death occurred in 4 patients after operation, giving a mortality rate of 6%. At operation, all 4 fatalities had both impacted ampullary stones and ASC, and 3 had bile peritonitis. Two patients died due to AOSC. A 61-year-old male had Parkinson's disease and died 345 days after operation because of pneumonia (Table 3).

*Eleven patients with hemorrhagic necrotizing pancreatitis (Group II):*
Four patients without complications of acute pancreatitis underwent either biliary surgery alone (3 cases) or biliary surgery together with pancreatic drainage (1 case). They experienced an uneventful postoperative course. Of the remaining 7 patients

**Table 3** Details of 4 fatalities in Group I

| *No.* | *Age (y) /Sex* | *Surgical indication* | *Findings at operation* | *Operative procedures** | *Cause of death* |
|---|---|---|---|---|---|
| 1. | 61/M | Peritonitis | Impacted ampullary stone<br>ASC<br>Bile peritonitis<br>Serous cholecystitis | (1) | Pneumonia<br>(345 PO) |
| 2. | 80/M | Shock | Impacted ampullary stone<br>ASC<br>Serous cholecystitis | (2) | AOSC<br>(1 PO) |
| 3. | 79/M | Peritonitis & shock | Impacted ampullary stone<br>ASC<br>Bile peritonitis<br>Gangrenous cholecystitis | (3) | AOSC<br>(1 PO) |
| 4. | 66/M | Peritonitis | Impacted ampullary stone<br>ASC<br>Bile peritonitis<br>Abscess around the GB<br>Serous cholecystitis | (3) | MOF<br>(62 PO) |

*: (1) cholecystectomy, choledocholithotomy, sphincterotomy and abdominal cavity drainage.
(2) cholecystectomy and choledocholithotomy.
(3) cholecystectomy, choledocholithotomy and abdominal cavity drainage.
ASC: acute suppurative cholangitis (purulent bile in the bile duct); AOSC: acute obstructive suppulative cholangitis; MOF: multi-organ failure; GB: gallbladder; PO: postoperative day.

with complications of acute pancreatitis, 2 in the early period of this study, a 54-year-old severely ill female and a 77-year-old female, who had extensive pancreatic necrosis with massive pancreatic ascites underwent biliary surgery and abdominal drainage. The former died of irreversible shock 3 days postoperatively and the latter died of multi-organ failure (MOF) caused by intraabdominal abscess 44 days after operation. The next 5 patients underwent operation for pancreatitis in conjunction with biliary surgery. The specific procedures for pancreatitis performed in these patients included abdominal, pancreatic and retroperitoneal drainage and/or abscess drainage (DRAINAGE) (3 cases) and necrosectomy in addition to DRAINAGE (last 2 cases). Of these, a 61-year-old male who had hemorrhagic pancreatitis and intraabdominal abscess and who underwent DRAINAGE died of MOF related to persistent intraabdominal abscess 112 days postoperatively. Overall, there were 3 postoperative deaths in Group II, giving a mortality rate of 27% (Table 4).

### CASE PRESENTATION

### *Case 1 (Group I)*

A 55-year-old female complained of severe abdominal pain. Laboratory data on admission was as follows: serum amylase 1890 (C.U.), SGOT 995 (K.U.), SGPT 715 (K.U.), T.Bil 2.7 (mg/dl) and WBC 18500. US demonstrated multiple stones in the dilated gallbladder. Conventional supportive treatment failed and her general condition deteriorated. Emergency operation revealed edematous pancreas and edematous gallbladder. Cholecystectomy was performed. Operative cholangiography demonstrated an impacted stone at the terminal bile duct, which was removed by transduodenal sphincterotomy, a stone about 8 mm in diameter was found lodged at the ampulla of Vater. After the stone was removed, pancreatic juice and purulent bile (ASC) gushed suddenly, and obstruction of both the pancreatic duct and bile duct was confirmed (Figure 1). Her postoperative course was uneventful.

### *Case 2 (Group II)*

A 68-year-old male complained of severe abdominal pain. The abdomen was distended and showed peritoneal irritation. Laboratory data on admission was as

**Table 4** Operative procedures and mortality in the 11 Group II patients

| *Operative procedures* | *Cases* | *Mortality* |
|---|---|---|
| Without complications of pancreatitis | 4 | 0 |
| (1): Biliary surgery | 3 | 0 |
| (2): (1) + Pancreatic drainage | 1 | 0 |
| With complications of pancreatitis | 7 | 3 (43%) |
| (3): (1) + Abdominal drainage | 2 | 2 (100%) |
| (4): (1) + DRAINAGE | 3 | 1 (33%) |
| (5): (4) + Necrosectomy | 2 | 0 |
| Total | 11 | 3 (27%) |

Drainage: abdominal, pancreatic and retro-peritoneal drainage and/or abscess drainage.

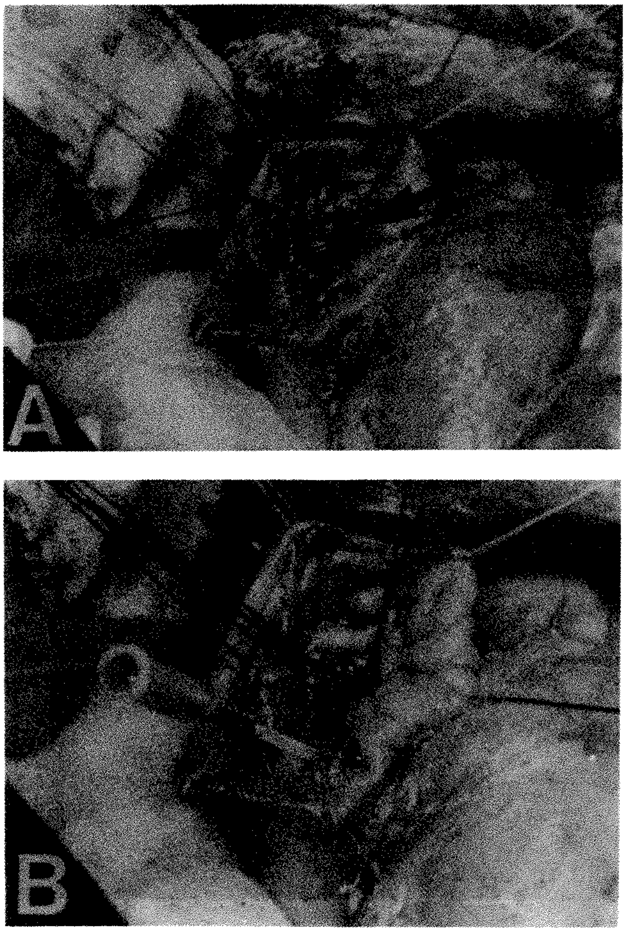

**Figure 1** Operative findings of case 1. A:At transduodenal sphincterotomy, a gallstone about 8 mm in diameter (arrow) was found lodged at the ampulla of Vater. B: After the stone was removed, pancreatic juice and purulent bile gushed suddenly, and obstruction of both the pancreatic duct and bile duct was confirmed. A venous cannula is inserted into the pancreatic duct.

follows: serum amylase 2213 (C.U.), SGOT 229 (K.U.), SGPT 88 (K.U.), T.Bil 1.1 (mg/dl) and WBC 15800. US showed multiple stones in the gallbladder, swelling of the pancreas and accumulation of fluid in the abdomen. Abdominal pain and peritoneal irritation did not settle by conventional medical therapy, and emergency operation was performed. Laparotomy revealed extensive pancreatic necrosis with massive pancreatic ascites (Figure 2). Operative cholangiography showed no bile duct stones. He received necrosectomy in addition to cholecystectomy and DRAINAGE. Emergency surgery relieved symptoms rapidly but a pancreatic fistula developed after operation (Figure 3).The fistula closed by suction drainage. After discharge from hospital, a pancreatic pseudocyst developed. This was treated successfully by cystojejunostomy during his second admission.

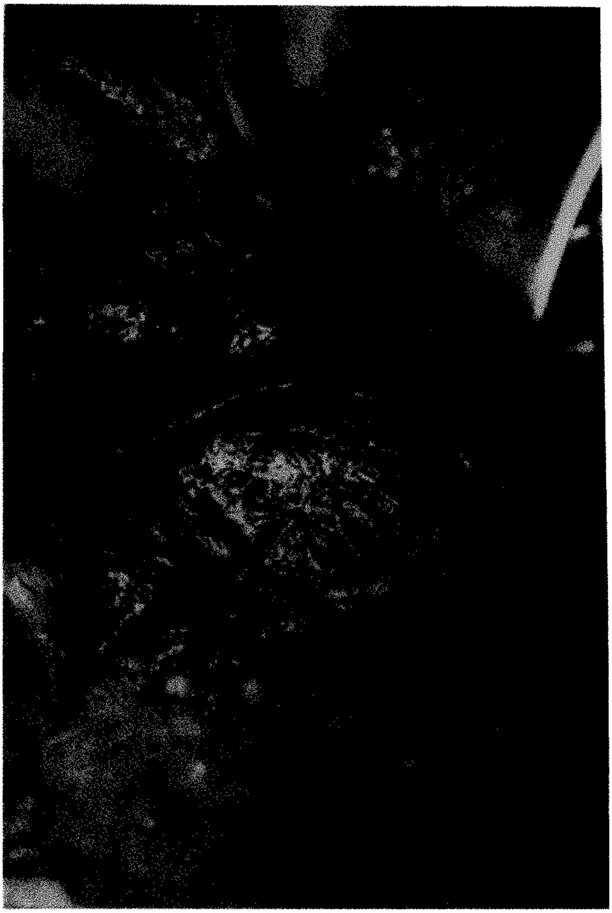

**Figure 2** Operative finding of case 2. Laparotomy revealed extensive pancreatic necrosis with massive pancreatic ascites. (*See colour plate at the back of this issue*).

### DISCUSSION

Acute pancreatitis is a disease characterized by severe abdominal pain and liberation of pancreatic enzymes into the blood stream. Gallstone associated pancreatitis is generally called biliary pancreatitis or gallstone pancreatitis. It has been reported, however, that 13–61%[4,5,7] of patients undergoing surgery within a few days of symptom onset lacked significant evidence of pancreatitis. Ranson[15] stated that some of the patients selected by the presence of abdominal pain, elevated amylase levels and demonstrated gallstones would include many with no evidence of pancreatitis, but with other illnesses such as cholangitis with hyperamylasemia or gallstone disease with hyperamylasemia, and that strict diagnostic criteria for assessing gallstones as the cause of pancreatitis might be necessary. In

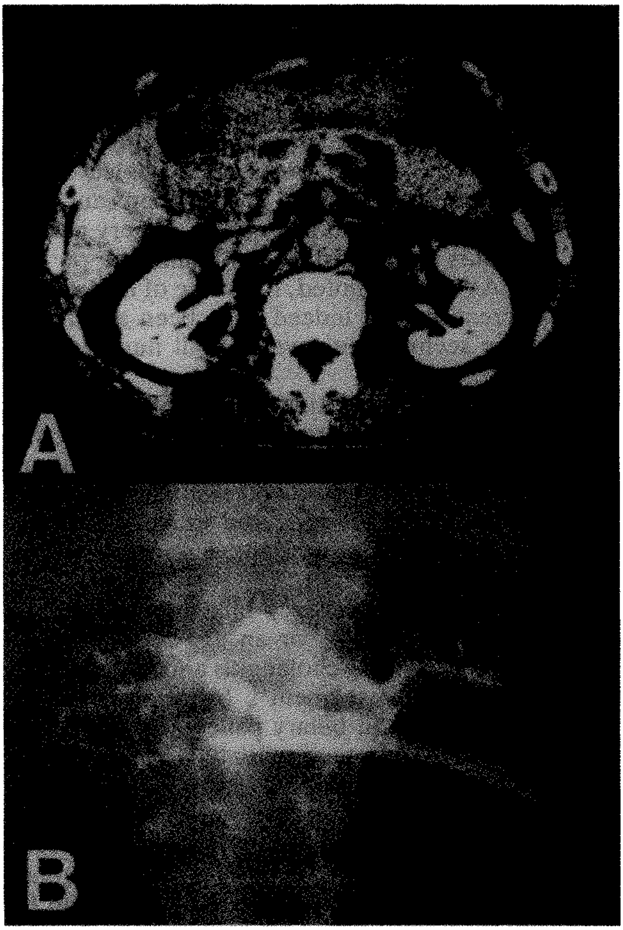

**Figure 3** Postoperative CT and fistulogram of case 2. A: Postoperative CT after contrast administration demonstrated a massive nonenhancing area in the body of the pancrease. B: After necrosectomy, a pancreatic fistula developed. Fistulogram shows accumulation of contrast material and outlines the pancreatic duct in the tail of the pancreas.

this respect, Acosta *et al.*[1] added the presence of jaundice to their criteria for biliary pancreatitis. McMahone and Pickford[11] reported that an increased plasma GOT level on the day of admission was suggestive of gallstones as the cause of pancreatitis. Meister[12] stated that elevation of serum amylase, bilirubin, SGOT and $\gamma$-GT would confirm a suspected biliary cause of the disease. Gossum *et al.*[13] reported that SGPT was the most discriminate test between biliary and nonbiliary pancreatitis.

Biliary pancreatitis is thought to be caused by the migration of bile duct stones into or through the ampulla of Vater[1,17], and the stones can lead to obstruction of both the bile and pancreatic duct[1,16] (obstructed common channel[17], Figure 1) or obstruction of the bile duct with a functioning common channel[17] during these transpapillary passage. Accordingly, we have added the presence of elevated serum transaminase levels in the early stage of biliary obstruction (gallstone hepatic injury or gallstone hepatitis[16]) as strict diagnostic criteria for assessing gallstone as the cause of pancreatitis. Whatever the mechanisms by which gallstones predispose patients to biliary pancreatitis, hepatic injury (gallstone hepatitis) and primary biliary tract disease (acute cholangitis and/or acute cholecystitis) caused by biliary obstruction must be ecomphasized (Figure 4). In the present series using such strict diagnostic criteria, 62 (85%) of the 73 emergency surgery patients who underwent operation within 48 hours of admission had minimal or mild pancreatitis at laparotomy (Group I). As Carter[18] noted, the cause of hyperamylasemia in patients with no apparent pancreatitis may indeed have been a mild attack of pancreatic inflammation, and there seem to be a very small number of patients that have hyperamylasemia alone[17].

The majority of the 62 Group I patients showed no objective improvement in response to conventional supportive therapy, 44 (71%) of whom had persistent life-threatening acute biliary tract disease including impacted ampullary stones, ASC, AOSC, bile peritonitis and gangrenous cholecystitis at laparotomy. Accordingly, their clinical features and symptoms appeared to stem mainly from acute biliary tract disease rather than pancreatitis itself, and emergency operation was necessary to correct the acute biliary tract disease.

In the 62 Group I patients, emergency biliary surgery was performed safely unless they had advanced age, AOSC or an associated medical problem.AOSC represents the end stage of ascending cholangitis and the operative mortality is high even with aggressive surgical management[14]. In recent years, ES has been advocated as an alternative approach in biliary pancreatitis[19]. In our hospital, ES had not been performed routinely for patients with biliary pancreatitis. Four patients in the present series underwent ES. Initial ES was unsuccessful in 2 of these patients and emergency operation was carried out to control sepsis[20]. However, in patients with advanced age, AOSC or medical contraindications to surgery, ES or percutaneous transhepatic biliary drainage (PTBD)[21] must be performed without delay.

Contrary to Group I patients, the majority of the 11 Group II patients who had more severe pancreatitis were uncomplicated by acute biliary tract disease. This could be explained by the passage of a stone, with rapid regression of acute inflammatory biliary disease. Just how the more severe pancreatitis occurs is the controversial area. Kelly *et al.*[3] reported that the progression of pancreatitis depends on the magnitude of the interaction between enzyme inhibitors and activated digestive enzymes. Duodenal regurgitation may occur through the open sphincter caused by the passed stones[17]. They also noted that the ultimate severity of an attack of pancreatitis is probably determined very early, perhaps even before signs and symptoms develop. Time between the onset of symptoms and operation in Group II patients in our series was short (1–5 days, mean 2.0 days).

The operative mortality of Group II was high (27%). The role of surgery in infected pancreatic necrosis and pancreatic abscess remains undisputed. Early surgery for patients with necrotizing pancreatitis and without infection, however, remains controversial and various forms of surgical treatment such as pancreatic drainage,pancreatic resection or debridement, peritoneal lavage, and thoracic duct drainage have been recommended[15]. Reasons for the high mortality in our series may have been due to failure to control the source of infection and ineffective surgical drainage. Based upon our recent experiences, drainage of intraabdominal,

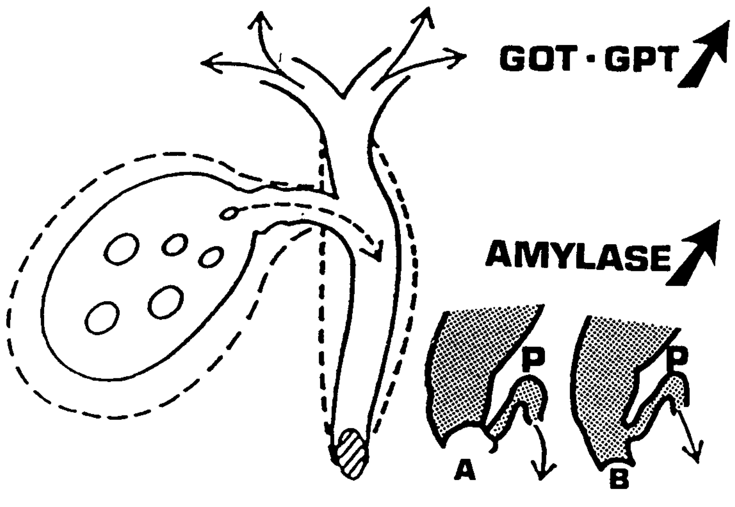

**Figure 4** Hepatic injury and biliary tract disease in biliary pancreatitis. Biliary pancreatitis is thought to be caused by the migration of stones into or through the ampulla of Vater, and the stones can lead to obstruction of both the bile duct and pancreatic duct (obstructed common channel[17], A) or obstruction of the bile duct with a functioning common channel[17] (B) during these transpapillary passage. Based upon the pathogenesis of biliary pancreatitis, hepatic injury (gallstone hepatitis[16]) and primary biliary tract disease caused by biliary obstruction must be emphasized. P: Pancreatic duct.

peripancreatic and retroperitoneal pancreatic fluid collections (DRAINAGE) might be useful to prevent systemic spread of biologically active compounds originating from the pancreas[22]. Necrosectomy may be the procedure of choice to prevent abscess formation before the necrotic tissue becomes a focus of infection[22]. In our series, the mortality rate of patients with hemorrhagic necrotizing pancreatitis complicated by pancreatic fluid collection and/or abscess was 100% in the 2 patients in the early period of this study who underwent biliary surgery and drainage of the abdominal cavity alone, 33% in the next 3 patients who underwent DRAINAGE together with biliary surgery and none in the last 2 patients who received necrosectomy in addition to DRAINAGE and biliary surgery. The number of patients included in the study is yet too small to draw conclusions, and large numbers of patients and randomized studies will resolve this question[23].

In light of our findings, the critical factor determining the type and timing of surgical intervention in biliary pancreatitis is not only the severity of the acute pancreatitis[23] but also the severity of the acute biliary tract disease. This is in keeping with the experience of Mackie[7] and Semel[8], who emphasized a pathological heterogeneity and coexistence of serious inflammatory biliary tract disease and pancreatitis, respectively. Because of the diversity of presentation, it is unlikely that any single management approach will be successful in all patients[8,23].

In conclusion, there appear to be two types of biliary pancreatitis refractory to conventional supportive therapy: (1) minimal or mild pancreatitis with persistent life-threatening acute biliary tract disease (biliary type) and (2) more severe pancreatitis (pancreas type). This classification is useful because the type and timing of surgical intervention and the mortality will vary between these two types of the disease. When patients with biliary pancreatitis fail to improve by conventional supportive therapy, precise and early discrimination between these two types of the disease is necessary. Clinical features such as Charcot's triad of acute cholangitis or paralytic ileus due to massive pancreatic ascites, impacted bile duct stones demonstrated by ERC, or PTC, and pancreatic changes on CT scan[24] are helpful in making the correct diagnosis.

### *Acknowledgements*

The authors are grateful to Yuji Nimura, M.D., Professor of First Department of Surgery, Nagoya University School of Medicine, for helpful criticisms.

### *References*

1. Acosta, J.M., Rossi, R., Galli, O.M.R., Pellegrini, C.A. and Skinner,D.B. (1978) Early surgery for acute gallstone pancreatitis: Evaluation of a systemic approach. *Surgery*, **83**, 367–370
2. Frey, C.F. (1987) A strategy for the surgical management of gallstone pancreatitis. *Acute Pancreatitis*. Berlin, Heidelberg, New York, London, Paris, Tokyo: Springer-Verlag
3. Kelly, T.R. and Wagner, D.S. (1988) Gallstone pancreatitis: A prospective randomized trial of the timing of surgery. *Surgery*, **104**, 600–605
4. Welch, J.P. and White, C.E. (1982) Acute pancreatitis of biliary origin: Is urgent operation necessary? *Am. J. Surgery*, **143**, 120–126
5. Kim, U., Shen, H.Y. and Bodner, B. (1988) Timing of surgery for acute gallstone pancreatitis. *Am. J. Surg.*, **156**, 393–396
6. Mayer,A.D., McMahon, M.J., Benson, E.A. and Axon, A.T.R. (1984) Operations upon the biliary tract in patients with acute pancreatitis: aims, indications and timing. *Ann. R. Coll. Surg. Engl.*, **66**, 179–183
7. Mackie, C.R., Wood, R.A.B., Preece, P.E. and Cuschieri, A. (1985) Surgical pathology at early elective operation for suspected acute gallstone pancreatitis: preliminary report of a prospective clinical trial. *Br. J. Surg.*, **72**, 179–181
8. Semel, L. Schrieber, D. and Fromm, D. (1983) Gallstone pancreatitis. *Arch. Surg.*, **118**, 901–903
9. Schwesinger, W.H., Page,C.P.,Sirinek,K.R.,Levine, B.A. and Aust,J.B. (1991) Biliary pancreatitis, operative outcome with a selective approach. *Arch. Surg.*, **126**, 836–840
10. Blamey, S.L., Imrie, C.W., O'Neill, J., Gilmour, W.H. and Carter, D.C. (1984) Prognostic factors in acute pancreatitis. *Gut*, **25**, 1340–1346
11. McMahon, M.J., and Pickford, I.R. (1979) Biochemical prediction of gallstones early in an attack of acute pancreatits. *Lancet*, **15**, 541–543
12. Meister, R. (1987) Treatment of biliary pancreatitis: Approach, technique, and results. *Acute Pancreatitis*. Berlin, Heidelberg, New York, London, Paris, Tokyo: Springer-Verlag
13. Gossum, A.V., Seferian, V.,Rodzynek, J.J., Wettendorff,P., Cremer, M. and Delcourt, A. (1984) Early detection of biliary pancreatitis. *Digestive Diseases and Sciences*, **19**, 97–101
14. Dow, R.W. and Lindenauer, S.M. (1969) Acute obstructive suppurative cholangitis. *Ann. Surg.*, **169**, 272–276
15. Ranson, J.H.C. (1990) The role of surgery in the management of acute pancreatitis. *Ann. Surg.*, **211**, 382–393
16. Isogai, M., Hachisuka, K., Yamaguchi, A. and Nakano, S. (1991) Etiology and pathogenesis of marked elevation of serum transaminase in patients with acute gallstone disease. *HPB Surgery*, **4**, 95–107
17. Kelly, T.R. (1984) Gallstone pancreatitis, Local predisposing factors. *Ann. Surg.*, **200**, 479–485
18. Carter, D.C. (1989) Gallstone pancreatitis. *Pancreatitis*. Edinburgh, London, Melbourne, New York: Churchill Livingstone
19. Neoptolemos, J.P. Carr-Locke, D.L., London, N.J., Bailey, I.A., James, D. and Fossarrd, D.P. (1988) Controlled trial of urgent endoscopic retrograde cholangiopancreatography and endoscopic sphincterotomy versus conservative treatment for acute pancreatitis due to gallstones. *Lancet*,. **ii**, 979–983
20. Himal, H.S. and Lindsay, T. (1990) Ascending cholangitis: Surgery versus endoscopic or percutaneous drainage. *Surgery*, **108**, 629–634

21. Pessa, M.E., Hawkins, I.F., and Vogel, S.B. (1987) The treatment of acute cholangitis. Percutaneous transhepatic biliary drainage before definitive therapy. *Ann. Surg.*, **205**, 389–392
22. Beger, H.G., Büchler, M., Bittner, R., Oettinger, W., Block, S. and Nevalainen T. (1988) Necrosectomy and postoperative local lavage in patients with necrotizing pancreatitis: Results of a prospective clinical trial. *World J. Surgery*, **12**, 255–262
23. Bradley, E.L. and Steinhause, E.P. (1991) Surgery in acute pancreatitis. The United States experience. *Int. J. Pancreatol.*, **9**, 67–73
24. Kivisaari, L., Somer, K., Standertsjold-Nordenstam, C.G., Schroder, T., Kivilaakso, E. and Lempinen M. (1984) A new method for the diagnosis of acute hemorrhagic necrotising pancreatitis using contrast-enhanced CT. *Gastrointest. Radiol.*, **9**, 27–30

*(Accepted by S.Bengmark 10 September 1992)*

### INVITED COMMENTARY

This paper from Japan addresses the nature of biliary pancreatitis. The authors consider that their patients can be divided into two groups namely those with mild pancreatitis but life threatening acute biliary tract disease and a group who develop more severe pancreatic inflammation early in the course of their disease. While it is generally accepted that some patients classified as having biliary pancreatitis turn out to have relatively mild inflammation if they come to laparotomy, my own feeling is that the paper is too rigid in its classification. In our practice there is undoubtedly a third group of patients who have pancreatitis that settles without going on to necrosectomy and its complications and in whom the biliary tract disease is not life threatening.

One of the striking features of this Japanese experience is indeed the large number of patients with severe biliary tract disease. No less than 34% of their 62 patients in Group 1 had Charcot's triad, 47% had acute suppurative cholangitis with or without gangrenous cholecystitis and 71% were thought to have life threatening biliary tract disease. Comparisons between this study and others are hampered by the failure to use objective scoring systems to assess the severity of the disease at presentation. The methods used to confirm the diagnosis are listed but we are not given any information regarding their detailed value. It is also surprising that only four patients had endoscopic retrograde cholangiograpy with attempted endoscopic shincterotomy. This would certainly be contrary to the practice in most centres although it must still be admitted that there is a lack of objective data to confirm the successful experience of the Leicester group in this context. It could be argued that many of the patients coming to urgent operation in this report from Isogai and colleagues would be spared the need for such surgery by appropriate use of endoscopic sphincterotomy.

I was also surprised to read that no less than 58 of the 62 patients in Group 1 had exploration of the common bile duct at the time of surgery. I accept that such exloration is indicated when stones are demonstrated on intraoperative cholangiography but would question the need for exploration when the only abnormality is failure of dye to flow freely into the duodenum. It is also recognised that some patients found to have stones in the duct on preoperative investigations will prove not to have stones at the time of surgery. These factors undoubtedly account for the fact that in 14 of the 62 cases (29%) stones were found only in the gall bladder at the time of operation.

In the Discussion, the authors support the use of drainage of intra-abdominal, peri-pancreatic and retro-peritoneal collections as a means of preventing systemic spread of biologically active compounds originating from the pancreas. Questions must be raised about the ability of drainage alone to deal adequately with problems in and around the pancreas following necrosectomy. The recent review by D'Egidio and Schein (*British Journal of Surgery*, 1991, 78, 133–137) indicates that this may be less satisfactory than the use of continuous lavage or open wound management in patients with these problems.

In conclusion, this paper is a useful addition to the literature in that it undoubtedly highlights the real problem of patients with biliary pancreatitis in whom it is the biliary tract disease which is life threatening. At the same time it raises important questions about the manner in which such patients should be managed but many will disagree with the need for urgent surgical intervention in many of the patients dealt with in this report.

David Carter
Prof. of Surgery
Edinburgh. UK

